# Case report: Exceptional and durable response to Radium-223 and suspension of androgen deprivation therapy in a metastatic castration-resistant prostate cancer patient

**DOI:** 10.3389/fonc.2024.1331643

**Published:** 2024-03-08

**Authors:** Francesca Zacchi, Joan Carles, Macarena Gonzalez, Xavier Maldonado, Raquel Perez-Lopez, Maria Eugenia Semidey, Joaquin Mateo

**Affiliations:** ^1^ Section of Innovation Biomedicine-Oncology Area, Department of Engineering for Innovation Medicine (DIMI), University of Verona and University and Hospital Trust (AOUI) of Verona, Verona, Italy; ^2^ Vall d’Hebron Institute of Oncology (VHIO), Barcelona, Spain; ^3^ Vall d’Hebron University Hospital, Barcelona, Spain

**Keywords:** Radium-223, bone response, bone metastases, metastatic castration-resistant prostate cancer, prognosis

## Abstract

Despite the development of new therapies in the last few years, metastatic prostate cancer (PCa) is still a lethal disease. Radium-223 (Ra-223) is approved for patients with advanced castration-resistant prostate cancer (CRPC) with bone metastases and no visceral disease. However, patients’ outcomes are heterogenous, and there is lack of validated predictive biomarkers of response, while biomarkers for early identification of patients who benefit from treatment are limited. This case report describes a remarkable and durable response to Ra-223 in a CRPC patient with bone metastases who had rapidly progressed to many previous therapies; this response is now lasting for 5 years even after having stopped backbone androgen deprivation therapy (ADT). Here, we present the clinical course of this exceptional response, as well as comprehensive genomic and histopathology analyses on sequential biopsies acquired before and after therapy. Additionally, we review current knowledge on predictive and response biomarkers to Ra-223 in metastatic prostate cancer.

## Introduction

PCa is one of the most common malignancies in men worldwide and significantly contributes, in the form of metastatic PCa, to increased mortality rates in men globally (1).

Systemic treatments represent the standard approach for metastatic diseases. PCa is driven by androgen receptor (*AR*)-signaling, making ADT with luteinizing hormone-releasing hormone (LHRH) agonists and *AR*-targeting drugs, such as abiraterone acetate or *AR* inhibitors, the mainstay of treatment for advanced PCa.

Beyond *AR* targeting therapy, taxane-based chemotherapy, and the advent of new drugs such as lutetium-177-PSMA (Prostate Specific Membrane Antigen), Ra-223, and Poly (ADP-ribose) polymerase (PARP) inhibitors, have improved life quality and overall survival (OS) in the setting of metastatic castration-resistant prostate cancer (mCRPC), but metastatic PCa remains a lethal condition (2–7).

Precision medicine, based on tailoring treatment strategies to the molecular make up of individual tumors, holds promise for improving metastatic PCa care; however, currently there are very few validated biomarkers to personalize treatment selection in advanced PCa (8, 9).

Bone is the most common site of metastasis in advanced PCa with a higher prevalence of osteoblastic, rather than osteolytic, metastases (10). These metastatic sites are frequently associated with pain and skeletal-related events, such as pathological fractures, spinal cord compression, or hypercalcemia, which reduce the quality of life and impact survival. The treatment of bone metastases in men with PCa is palliative with the goals to improve survival, relieve pain, improve mobility, and prevent complications (11). Nevertheless, the standard-of-care imaging techniques [e.g., computed tomography (CT) and bone scan (BS)] fail to give an accurate assessment of the burden of bone metastases, as well as to monitor changes in response to treatment; at present, there are no established radiographic criteria for response to systemic therapy in bone disease (12, 13).

Recent studies have shown the high diagnostic performance of new imaging techniques as diffusion-weighted MRI (Magnetic Resonance Imaging) and PSMA-PET (positron emission tomography) to detect bone metastases, exploring their role as response biomarkers during anticancer therapy, but further prospective evaluations are needed (14–16).

Moreover, another important challenge in the treatment of bone metastatic PCa is the different tumour microenvironment (TME) compared with that of soft tissue metastases, resulting in a suboptimal response of bone metastatic CRPC to immune checkpoint therapy (ICT) (17, 18). Recent evidence, as the TGF–β (Transforming growth factor-beta) involvement in the restraining development of Th1 (T-helper type 1) subset or the increased myeloid and neutrophil immune subsets in bone microenvironment after ICT therapy among others, suggest the strategy of targeting the microenvironment associated with bone metastases to improve anti-tumour responses with ICT, leaving this field an open challenge for the future.

Ra-223 is a targeted alpha emitter that acts as a calcium mimetic, forming complexes with hydroxyapatite and selectively binding areas of increased bone turnover such as osteoblastic bone metastases. The alpha particles, with a short path length in tissue and a high linear energy transfer, allow Ra-223 to kill an increased number of cells in its target tissue sparing the surrounding normal tissues (19, 20). In particular, the high-energy alpha-particle radiation induces mainly DNA double-stranded breaks (DSBs) that result in a potent and highly localized cytotoxic effect in the target areas (21). To date, Ra-223 therapy is approved in metastatic CRPC patients with symptomatic bone metastases and no visceral metastases or malignant lymphadenopathy larger than 3cm, while its role in asymptomatic patients is still not well defined (22, 23).

Here, is presented the case of a metastatic CRPC patient affected by few and oligosymptomatic bone metastases, with an exceptional and durable response after 5 years from the starting of Ra-223 therapy and a concomitant suspension of ADT. The patient is still clinically asymptomatic with a persistent radiological response and undetectable PSA (Prostate specific antigen) levels.

## Case report

A 65-years-old man with an Eastern Cooperative Oncology Group Performance Status 0 (ECOG-PS) without comorbidities was diagnosed with localized PCa and underwent a laparoscopic radical prostatectomy plus a lymphadenectomy in January 2014, confirming an adenocarcinoma, characterized by a Gleason Score (GS) of 9 (4 + 5), pT2N0 (0/14 nodes positive), with affected surgical margins.

**Figure 1:**
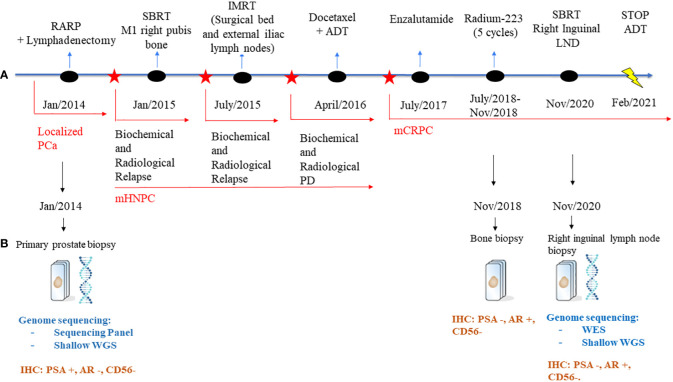
Schematic representation of the patient’s treatment history. **(A)** Treatment timeline. **(B)** Genome sequencing and IHC performed on the biopsies throughout the disease course. PCa, Prostate Cancer; RARP, Radical prostatectomy; ADT, adrenal deprivation therapy; PD, progression disease; mHNPC, metastatic hormone naïve prostate cancer; mCRPC, metastatic castration-resistant prostate cancer; LNDs, lymph nodes; NGS, Next generation sequencing; WES, whole genome sequencing; WGS, whole genome sequencing; IHC, immunohistochemistry.

Next generation targeted sequencing (NGS) of the diagnostic biopsy shows pathogenic/likely pathogenic alterations in *PTEN* (exon8:c.968dupA:p.N323fs), *TP53* (exon6:c.583A>T:p.I195F) with associated *TP53* deletion, *AASXL1* (exon12:c.2831dupT:p.L944fs) and *FANCL* (exon14:c.1096_1099dup:p.T367fs ***)*** (**Figure 1**).

Eleven months after surgery, the patient presented biochemical relapse with a PSA doubling time of 2.5 months; a 18 F-choline PET/CT scan revealed a single metastasis in the right pubis bone for which in January 2015 Stereotactic Body Radiation Therapy (SBRT) was administered (25 Gy in 3 fractions,fr). Nevertheless, the PSA continued to rise and in June 2015 a new 18 F-choline PET/CT scan showed a local relapse on the surgical bed and on external iliac lymph nodes for which received an Intensity-modulated radiation therapy (IMRT with a total dose of 70 Gy) on the sites of relapse.

**Figure 2:**
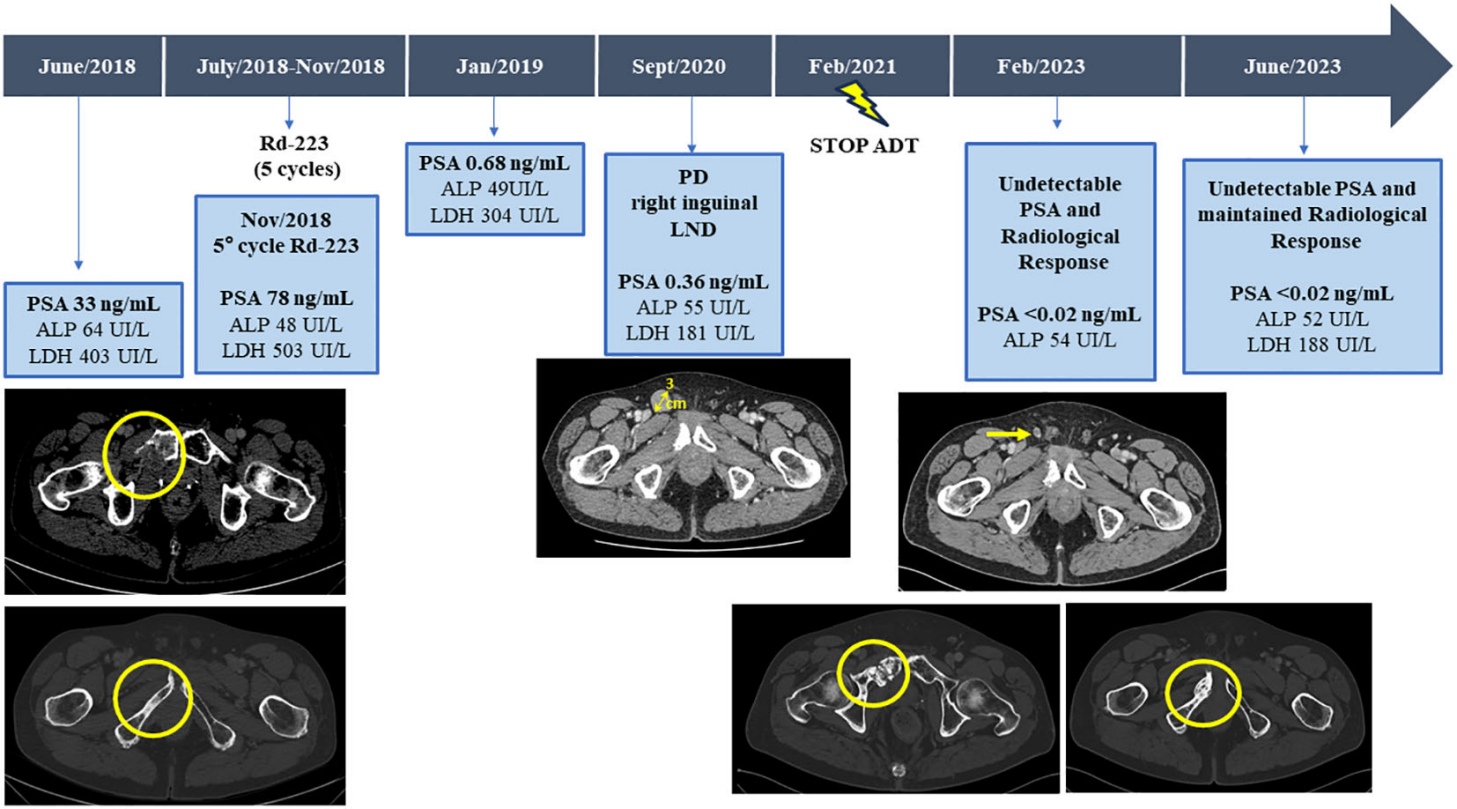
Serum bone biomarkers values (ALP, LDH, and PSA) and CT scans before and after third line with Radium-223 treatment, throughout the disease course. ALP, alkaline phosphatase; LDH, lactic acid dehydrogenase; PSA, Prostate-specific antigen; CT, computed tomography.

In March 2016, the patient presented a biochemical progression without radiological relapse on 18 F-choline PET/CT scan but with the evidence of two small new bone metastases (D10 and left ischiopubic bone metastases) on whole-body MRI. Because of this metastatic spread, the patient started a systemic treatment with ADT and Docetaxel (baseline PSA: 6.023 ng/dL), completing 6 cycles of chemotherapy and achieving ten months after the end of chemotherapy a complete biochemical and radiological response. However, the patient progressed to a metastatic CRPC status by biochemical criteria in June 2017 (PSA 8.32 ng/mL; testosterone 0.13 ng/dL) and started treatment with the *AR* inhibitor enzalutamide, achieving as best response stable disease by Response Evaluation Criteria in Solid Tumours (RECIST) version 1.1 (12), but with continuous rising PSA. Due to lack of symptoms, he continued enzalutamide until radiographic progression after 11 months of treatment. Progression was documented with new bone metastases confirmed on Bone and CT scans: an osteolytic bone metastasis with a soft tissue component in the right ileopubic ramus and an osteoblastic bone metastasis in the right ischiopubic ramus (**Figure 2**).

Therefore, with a baseline serum PSA of 33ng/mL, alkaline phosphatase (ALP) of 64UI/L and lactic acid dehydrogenase (LDH) of 403UI/L, the patient started a third line with Ra-223 as part of clinical trial exploring the role of Ra-223 in asymptomatic or pauci-symptomatic patients (EXCAAPE phase II trial) from July 2018 to November 2018. While the possibility of external Beam Radiation Therapy (EBRT) was evaluated, Ra-223 was finally prioritized due to accumulated exposure to radiation in the pelvis from the prior treatments in 2015 and the rapid PSA kinetics. Before starting Ra-223, the patient reported grade 1 lumbar pain as the only symptom, not requiring of painkillers. After the first cycle of Ra-223, he reported acute pain in right hemipelvis, which limited walking for a few days and then improved. The same pattern occurrent after each of the Ra-223 administrations, with temporary increases in pain, compromising mobilization, in the region of the right hemipelvis bone metastasis and concomitant increases in LDH and PSA but stable ALP (PSA 33ng/mL, ALP 71UI/L and LDH 518UI/L before the second cycle of Ra-223; PSA 37ng/mL, LDH 455UI/L and ALP 55UI/L before the third cycle; PSA 47ng/mL, LDH 594 UI/L, ALP 48 UI/L before the fourth cycle and PSA 78ng/mL, ALP 48UI/L and LDH 503UI/L before the fifth cycle). Due to increased pain and rising PSA, new imaging tests were performed after the third cycle of Ra-223, showing stable disease on bone scan but a cortical fracture on the lytic ileopubic lesion with associated soft tissue component on the CT scan.

After 5 cycles of Ra-223, and in the context of increasing pain, it was agreed with the patient to terminate therapy and not to administer the planned 6^th^ cycle of Ra-223. Finally, the bone pain was controlled with non-steroidal anti-inflammatory drugs (NSAIDs), not requiring further radiation therapy.

**Figure 3:**
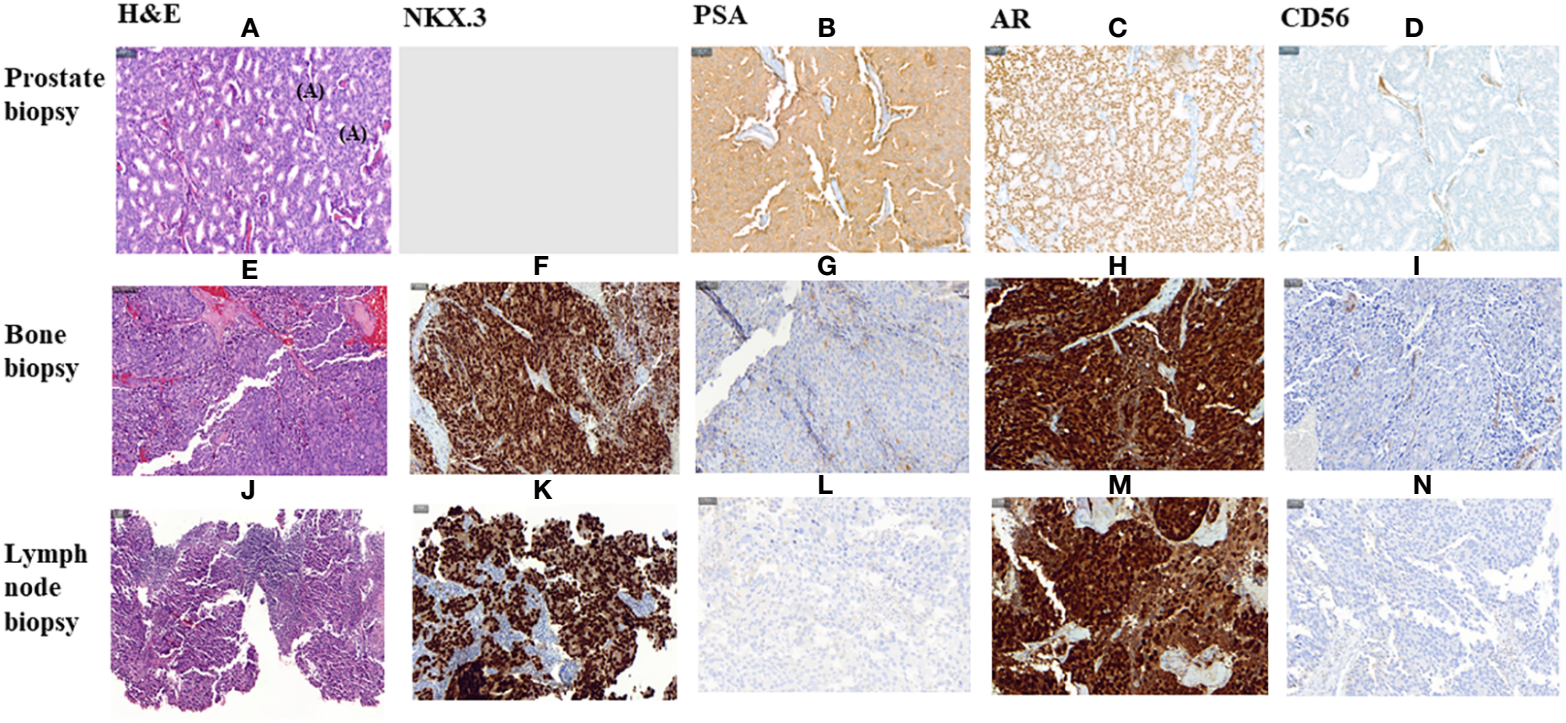
Histological morphology on H&E stains and IHC characteristics of FFPE section of a primitive prostate biopsy, metastatic bone biopsy and metastatic lymph node biopsy from Prostate Cancer adenocarcinoma. **(A–I, K–N)**. magnification 200X. **(J)** magnification 100X. HE, Haematoxylin and Eosin; IHC, Immunohistochemistry; PSA, Prostate specific antigen; *AR*, Androgen Receptor.

In January 2019, three months after finishing therapy with Ra-223 and with no other intercurrent therapies, the patient underwent a restaging assessment with a bone and CT scans showing stable disease and an unexpected decrease of PSA (0,68 ng/mL) and started radiological and biochemical follow-ups with a progressive decline of the PSA until a nadir of 0,1 ng/mL in April 2019. A biopsy on the right ileopubic bone lesion was performed prior to the imaging evaluation, confirming the presence of prostate adenocarcinoma cells, with a strong expression in IHC (immunohistochemistry) of Androgen receptor (*AR*) and NKX3.1, a weak expression of PSA and the negativity of neuroendocrine markers (Chromogranin A, CD56) (**Figure 3**).

The patient continued follow-up visits. A few months later, an osteoporotic fracture in the L5 vertebral body was diagnosed, with no signs of metastatic disease by MRI at that location, and the patient started bisphosphonates (Zometa 4mg every three months).

The PSA values remained <1 during 2019 and 2020, and the patient was mostly asymptomatic. In September 2020, an abnormal right inguinal lymph node was noted at the physical exam at one of the regular follow-up visits, with a PSA of 0,36 ng/mL. CT and bone scan were performed, and the only abnormality beyond the residual images in the bone disease was this palpable 3 cm right inguinal lymph node; an US (ultrasound)-guided biopsy of the node was performed, confirming the diagnosis of metastasis of prostate adenocarcinoma. IHC assessment of the lymph node revealed a strong expression of *AR* and NKX3.1, and weak expression of PSA (in line with the low PSA levels in serum) and negativity for neuroendocrine markers Chromogranin A and CD56 (**Figure 3**). Whole-exome sequencing of the lymph node biopsy shows important *AR* amplification (66 copies) and *MYC* and *FOXA1* amplification, as well as *PTEN* and *FANCA* deletions. The original *TP53* mutation detected in the primary tumor (p.I195F) was also detected, confirming the common origin (**Figure 1**).

In November 2020, the patient received radiotherapy on the local lymph node progression (35Gy in 8 fractions), achieving a partial radiological response in the follow up scans (<1cm diameter) with no other evidence of extra-osseus disease; PSA during 2021 remained low (PSA nadir <0.02 ng/mL).

Two and a half years after the last cycle of Ra-223, the patient decided to stop ADT in February 2021, due to concerns about osteoporosis and cardiovascular risks.

Despite the suspension of ADT therapy, 5.5 years after the start of Ra-223 therapy the patient remains asymptomatic, with a maintained radiological response, with no PSA detection in blood (PSA <0.02 ng/mL) and normalization of other disease-related biomarkers (ALP 52UI/L and LDH 188UI/L).

## Discussion

Ra-223 is a life-prolonging agent in patients affected by bone-metastatic CRPC but many open questions remain about its use in clinical practice. In the registration phase III trial (ALSYMPCA), symptomatic bone metastatic CRPC patients who have received best standard of care were treated with Ra-223 and showed a significantly better OS than the placebo group, achieving a median OS of 14.9 months vs 11.3 months (HR 0,70, 95%CI 0,58-0,83). However, the benefit was unclear in the subgroup of patients with less than six bone metastases (23), such as the case presented here. The use of Ra-223 in oligo or asymptomatic patients is further supported by results from international Expanded access program (iEAP), and from a phase II study of our group in asymptomatic patients who progress after first line hormonal therapy in metastatic CRPC demonstrating a PFS of 5.5 months (24, 25).

The optimal time for using this therapy during is a matter of debate since the increase skeletal related events observed in the ERA223 trials (26). Ra-223 is a bone-targeting agent that mimics calcium ions and is adsorbed at bone sites of active mineralization and high osteoblastic turnover (20). For this reason, Ra-223 principally targets PCa metastatic foci where osteoblasts surround PCa cells but can also accumulate in other locations with high bone turnover, such as degenerative bone disease (osteoporosis) or recently (micro)fracture, increasing the risk of fracture.In the case here presented, however, the patient presented a mixture of blastic and lytic metastasis, but the response to therapy was probably homogeneous among them, considering none of the lesions has relapsed after 5 years. This highlights the need for further research for assessing response and progression in bone lesiones, which are limited with current standard-of-care imaging techniques, as well as on the biology of bone metastasis and them mechanisms of action of this agent.

The risk of osteoporosis in metastatic CRPC patients is already increased using continuous ADT. In this case, the patient decided to stop ADT after 3 years of maintained response. While continuous ADT remains the standard-of-care among metastatic CRPC patients, one could argue the role of ADT in patients with exceptionally prolonged responses and no evidence of residual disease is unclear. While these cases are uncommon, and there is no clear agreement upon how to define an “exceptional response”, the fact that the disease has not returned after 2.5 years off-ADT is provoking and goes in line to current research aiming to minimize the impact of testosterone suppression among men with prostate cancer (27, 28). Recent results from the EORTC1333/PEACE III trial (29, 30) have shown that the risks of osteoporosis associated to ADT and Ra-223 can be controlled with appropriate use of bisphosphonates. Therefore, is important to assess the state of bone health and patient’s baseline fracture risk before starting treatment with Ra-223 to evaluate the risk benefit ratio and preventive measures such as the use of VitD, Calcium supplementation, Bisphosphonate or Denosumab.

An important challenge with Ra-223 therapy is response assessment, considering the predominantly bone disease. Indeed, the RECIST version 1.1 do not define progression or response of bone metastases, as are considered non measurable disease, and Prostate Cancer Clinical Trials Working Group 3 (PCWG3) defines criteria for bone progression on bone scan but fails to establish criteria for bone response assessment (12, 13). Considering Ra-223 is used in late-stage prostate cancer, early readouts of patient benefit are needed to avoid prolonged exposure to inefficient therapies. Decrease of serum PSA is not necessarily expected during Ra-223 therapy and do not seem to be a good marker of patient benefit; this could be explained by the Ra-223 mechanism principally directed towards the bone microenvironment then to the PCa cells directly. In this case, the patient experienced a PSA rise on therapy concomitantly to increased pain, followed by a profound drop in PSA after therapy; this was possible secondary to the lack of PSA expression in his *AR* positive metastatic biopsies, and not necessarily translating antitumor effect directly. According to the literature, generally low PSA secretors present small cell neuroendocrine features with lower *AR* transcriptional signature scores. However, this case presented strong expression of *AR* and NKX3.1, and no genomic loss of KLK3 (the gene encoding for PSA) was detected. Whether this was the result of subclonal selection, or post-genomic mechanism inactivating PSA translation remains unclear in this case (31, 32).

Overall, this further supports that PSA levels are not an appropriate marker to monitor response to Ra-223. Conversely, high basal total level of serum ALP and LDH prior Ra-223 treatment and the correlation with worse outcomes in OS and PFS, suggest their rule as prognostic biomarkers; In addition, the relationship between ALP levels changes and OS during Ra-223 treatment suggest its rule as a response biomarker in metastatic CRPC with bone metastases during Ra-223 (33). Further research into novel imaging techniques such as diffusion-weighted MRI or PET imaging, or other biomarkers such as CTC (circulating tumor cells) or cell-free DNA burden in circulation, to inform clinical decisions when using Ra-223 is also needed (34–36).

There is also a lack of validated predictive biomarkers to identify men who are more likely to derive significant benefit from Ra-223 therapy. Since the induction of DNA damage is the major mechanism of action of Ra-223, it has been hypothesized that Ra-223 could be more effective in patients with genomic defects in DNA repair genes (37, 38). Multiple retrospective studies have explored the efficacy of Ra-223 in metastatic CRPC patients with mutations in DNA repair genes (39–42). Overall, a trend towards more favorable ALP response rates, more durable responses and a more favorable OS figures has been observed in this molecularly defined subset of prostate cancer, but conclusive data is missing, and no prospective validation has been conducted. The case here reported presented loss of the *FANCA* gene, but the impact of this event in drug sensitivity in unclear. No other alterations in DDR genes were detected in either of the two analyzed biopsies. In these NGS analysis of the post-Ra-223 bone biopsy and lymph node relapse, the main findings were *AR* amplification, in line with prior use of hormonal therapies, and loss-of-function events in *TP53* and *PTEN*, commonly associated to poor prognosis in prostate cancer, which makes this exceptional response even more remarkable. Indeed, the patient had before experienced relatively short progression-free survival to ADT, docetaxel, and enzalutamide.

## Conclusion

Ra-223 represents a therapeutic option within the wide therapeutic armamentarium for the metastatic CRPC patients with bone metastases.

This case presented with few and oligosymptomatic bone metastases and derived exceptionally prolonged benefit from Ra-223 monotherapy allowing even for ADT discontinuation and remaining disease-free after 5 years. This case underscores the lack of validated tools to guide clinical decisions when using this drug, stressing the need for further research on imaging and molecular biomarkers to identify patients who may benefit from Ra-223 treatment, aiming to ultimately improve quality of life and survival of metastatic CRPC patients.

## Data availability statement

The raw data supporting the conclusions of this article will be made available by the authors, without undue reservation.

## Ethics statement

The studies involving humans were approved by Ethics Committee for Clinical Research at the Vall d’Hebron Hospital Ethics Committee. The studies were conducted in accordance with the local legislation and institutional requirements. The participants provided their written informed consent to participate in this study. Written informed consent was obtained from the individual(s) for the publication of any potentially identifiable images or data included in this article.

## Author contributions

FZ: Writing – original draft. JC: Writing – review & editing. MG: Writing – review & editing. XM: Writing – review & editing. RP-L: Writing – review & editing. MS: Writing – review & editing. JM: Writing – original draft, Writing – review & editing.
